# Alteration of perivascular reflectivity on optical coherence tomography of branched retinal vein obstruction

**DOI:** 10.1038/s41598-023-41691-4

**Published:** 2023-09-22

**Authors:** Bo-Een Hwang, Joo-Young Kim, Rae-Young Kim, Mirinae Kim, Young-Geun Park, Young-Hoon Park

**Affiliations:** 1 Department of Ophthalmology and Visual Science, Seoul St. Mary’s Hospital, College of Medicine, The Catholic University of Korea, 222 Banpo-daero, Seocho-gu, Seoul, 06591 Republic of Korea; 2 Catholic Institute for Visual Science, College of Medicine, The Catholic University of Korea, Seoul, Republic of Korea

**Keywords:** Retinal diseases, Vision disorders

## Abstract

This study aimed to evaluate perivascular reflectivity in patients with branched retinal vascular obstruction (BRVO) using en-face optical coherence tomography (OCT). The study retrospectively analyzed 45 patients with recurrent BRVO, 30 with indolent BRVO, and 45 age- and sex-matched controls. Using a 3.0 × 3.0-mm deep capillary plexus slab on macular scans, OCT angiography (OCTA) and structural en-face OCT scans were divided into four quadrants. Obstructive quadrants of OCTA scans were binarized using a threshold value of mean + 2 standard deviation. The selected area of high signal strength (HSS) was applied to the structural en-face OCT scans, and the corrected mean perivascular reflectivity was calculated as the mean reflectivity on the HSS area/overall en-face OCT mean reflectivity. The same procedure was performed in the quadrants of the matched controls. Regression analysis was conducted on several factors possibly associated with corrected perivascular reflectivity. The perivascular reflectivity in the obstructive BRVO quadrant was significantly higher than in the indolent BRVO and control quadrants (P = 0.009, P = 0.003). Both univariate and multivariate regression analyses showed a significant correlation between the average number of intravitreal injections (anti-vascular endothelial growth factor or dexamethasone implant) per year and refractive errors and image binarization threshold and perivascular reflectivity (P = 0.011, 0.013, < 0.001/univariate; 0.007, 0.041, 0.005/multivariate, respectively). En-face OCT scans of the deep capillary plexus slab revealed higher perivascular reflectivity in recurrent BRVO eyes than in indolent BRVO and control eyes. The results also indicate a remarkable correlation between perivascular reflectivity and the average number of intravitreal injections, suggesting a link to recurrence rates.

## Introduction

Branched retinal vascular obstruction (BRVO) can cause permanent vision loss when accompanied by recurrent or persistent clinical macular edema (ME). Intravitreal injections of anti-vascular endothelial cell growth factor (VEGF) or steroids are currently being used to resolve ME and prevent recurrences^1,2^. However, in cases where recurrent ME does not respond well to these treatments, ophthalmologists should investigate the underlying causes of recurrence because repeated intravitreal injection treatments can be a financial burden to patients and result in irreversible vision loss. In addition, early intensive care has been shown to affect ME recurrence and visual prognosis^3–5^. Therefore, identifying patients at risk of recurrent ME early and providing initial intensive intravitreal injection treatments are of great significance.

Risk factors for ME recurrence have been extensively studied clinically and experimentally. Recent reports indicate that the prognosis varies depending on the degree of ischemia (perfusion) or vessel congestion on optical coherence tomography (OCT) angiography (OCTA) and fluorescent angiography^6,7^. Other studies have reported changes in cytokine or protein concentrations in the aqueous humor involved in recurrence^8–10^. Considering that the pathophysiology of BRVO ME is mainly explained by the blood–retinal barrier (BRB) breakdown, local changes in retinal blood vessels and the surrounding tissues that form the BRB may be one of the most important factors in predicting ME recurrence.

Structural OCT reflectivity has been reported on the association with clinical features and visual prognosis in ischemic retinopathy^11,12^. However, no study has analyzed the relationship between changes in reflectivity around blood vessels where ME occurs and the clinical prognosis, particularly concerning the number of intraocular injections administered to treat recurrences. OCTA parameters should be compared with clinical patterns by measuring the degree of ischemia or perfusion and confirming the collateral vessels^13–15^.

In the present study, we analyzed reflectivity in the perivascular area of en-face OCT and investigated its relationship with potential prognostic factors in patients with BRVO ME.

## Methods

### Study population

This retrospective observational study was conducted at the Department of Ophthalmology and Visual Science in Seoul St. Mary's Hospital, The Catholic University of Korea, and adhered to the tenets of the Declaration of Helsinki. All protocols were approved by the Institutional Review Board (IRB) of Seoul St. Mary’s Hospital. Owing to the retrospective nature of this study and the use of anonymized data, waiver of informed consent was granted by the IRB in accordance with the provisions of the IRB.

Seventy-five patients diagnosed with monocular BRVO at our clinic were included in our study, along with forty-five sex- and age-matched control eyes. All the participants were selected between September 2020 and August 2021 at Seoul St. Mary's Hospital. A retrospective review of medical records was conducted, and exclusion criteria were applied as follows: (1) refractive errors of exceeding ± six diopters (spherical equivalent); (2) eyes with a history of ocular trauma, laser treatment, or intraocular surgery; (3) eyes with a history of intravitreal injections for other ocular diseases; (4) other systemic diseases that could affect the retina, except hypertension and diabetes mellitus; (5) other retinal diseases, including glaucoma, age-related macular degeneration, diabetic retinopathy, neurodegenerative disease, or other retinal diseases affecting the macular lesion; (6) media opacity that could affect image quality; and (7) any history of uveitis.

### Study protocol

**Figure 1:**
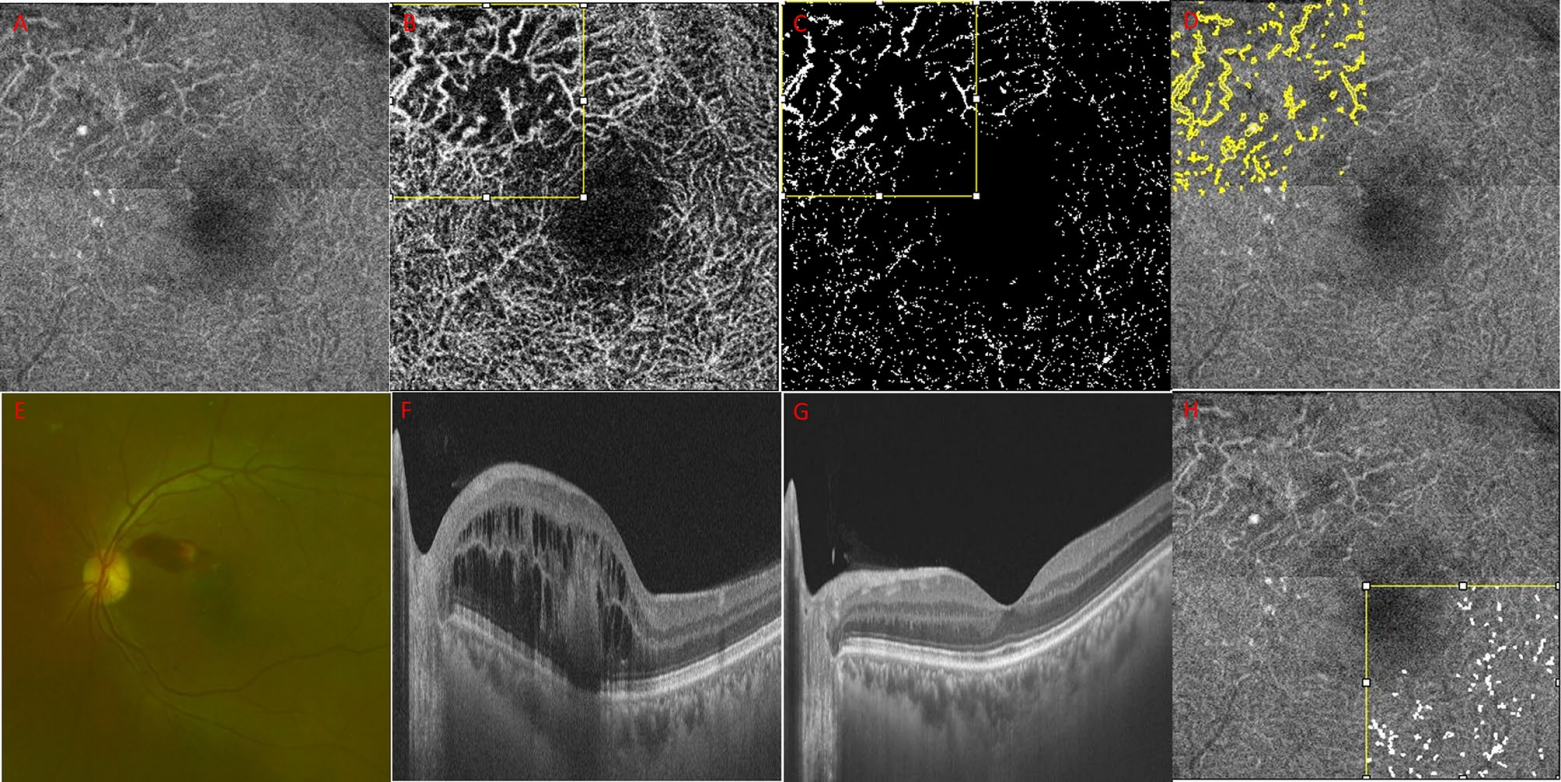
A representative case of the perivascular reflectivity calculation using deep capillary plexus slab en-face structural optical coherence tomography (OCT) and OCT angiography (OCTA) images. (**a**,**b**) The 3.0 × 3.0-mm (320 × 320 pixels) deep capillary plexus slab on en-face structural OCT and OCTA images were obtained using the OCTARA segmentation algorithm integrated into the Topcon imageNET software. (**b**,**c**) Binarization with a threshold of mean pixel value + 2 standard deviation (SD) was applied to the branched retinal vascular obstruction (BRVO)-affected quadrant of the OCTA image. (**c**,**d**) The vessel area with the prominent flow was calculated using the “analyze particle” Image J tool (> three pixels) on OCTA and presented as a yellow-colored area on the structural OCT image. (**e**,**f**) Fundus photography and OCT of the active BRVO stage with macular edema at the initial visit. (**g**) State of the resolved macular edema when perivascular reflectivity calculation was conducted (> 6 months). (**h**) Perivascular reflectivity of the BRVO contralateral quadrant was measured using the same method for comparison with the obstructive quadrant.

During the initial visit, demographic data, medical history, and ophthalmologic history were recorded. All participants underwent ocular examinations, including slit-lamp microscopy, dilated fundus examination, OCT, and OCTA. OCT and OCTA were performed continuously using the Topcon DRI Triton SS-OCT device with a 1050-nm wavelength light source and a scanning speed of 100,000 A-scans/s. BRVO was diagnosed when its typical characteristics (i.e., regional flame shift hemorrhage along with vessels and ME) were present on fundus examination and OCT images (as shown in Fig. 1e,f). BRVO eyes with vitreous hemorrhage and multiple vascular obstructions (e.g., hemi–central retinal vein occlusion) and eyes with major BRVO that did not affect macular lesions were excluded. All patients received intravitreal injection treatment to resolve ME. According to the injection protocol, anti-VEGF injections were administered to ME with intermittent usage of dexamethasone implants in unresponsive cases. The BRVO group (n = 75) was divided into recurrent and indolent BRVO subgroup. Patients who acquired a state of resolved ME without an intravitreal injection or with 1 to 2 injections in > 3 years were assigned to the indolent BRVO subgroup. Patients who received multiple intravitreal injections or developed persistent macula edema were assigned to the recurrent BRVO subgroup. On the basis of reports suggesting a high correlation between deep capillary plexus (DCP) lesions and ME and visual acuity^16^, 3.0 × 3.0-mm en-face OCT DCP macular scans were selected for analysis at the time of resolved ME, at least 6 months after the initial visit (Fig. 1g). The scan at the time of resolved ME was chosen to prevent the intraretinal fluids from affecting the OCT and OCTA signal strengths. Two experienced independent retinal specialists (Y-H.P. and B-E.H.) who were blinded to the other imaging findings and clinical histories evaluated all en-face OCT and OCTA images.

### En-face structural OCT perivascular reflectivity measurement on the area of high signal strength on OCTA

All en-face OCT images in this study met a minimum image quality of 65, with no line artifacts or noise. Figure 1 shows the process of selecting a high signal strength (HSS) area on OCTA and calculating the perivascular reflectivity of the corresponding area in the en-face structural OCT images. The 3.0 × 3.0-mm (320 × 320 pixels) DCP images were obtained using the DCP slab, which was calculated using the OCTARA segmentation algorithm built into the Topcon imageNET software (Fig. 1a,b). The images were divided into four quadrants, and two quadrants were selected for comparison to identify regional differences: the obstructive and contralateral quadrants. Binarization with a threshold of mean pixel value + 2 standard deviations (SDs) was applied to the OCTA images (Fig. 1b,c)^17^. We used the “analyze particle” command in the Image J software (version 1.53a; https://imagej.nih.gov/ij/)^18^ to calculate all white particle–like objects bigger than three consecutive pixels (approximately 28 μm) on the OCTA images to designate the area of HSS. The HSS area was then applied to the corresponding structural en-face OCT images, and the perivascular mean reflectivity was measured in that area (Fig. 1d,h). We calculated the mean corrected perivascular reflectivity as the mean reflectivity on the HSS area/overall en-face OCT mean reflectivity. We repeated the same procedure for the quadrants of the matched controls. We chose to designate HSS as a continuous particle of three pixels or more because the lateral resolution of swept-source OCT (SS-OCT) used in this study was 20 μm and the size of the retinal arterioles and venules was 15 μm or larger. This approach minimized signal noise and provided a measurable appropriate size for the signal of three pixels or more.

On OCTA, the area in which blood flow is measured displayed brightly, and the final brightness (reflectivity) in a selected scan was determined by accumulating decorrelation signals of each scan depth^19^. The HSS area represented a blood-rich area in which a detection of blood flow overlapped. We calculated reflectivity only in the HSS area, which contained pixels larger than mean pixel values + 2 SD of each scan, because analyzing the reflectivity in an area in which blood flow is definite and abundant effectively reduces the confounding factors for structural en-face OCT reflectivity.

### Choroidal thickness, foveal avascular zone parameter, deep capillary plexus vessel density, fluorescein angiography nonperfusion area measurement

Choroidal thickness (CT) was determined using the automatic built-in software within the SS-OCT device. Subfoveal CT was calculated by measuring the distance from the outer border of the RPE to the inner edge of the suprachoroidal space^20^. The CT at the foveal center was manually measured using digital calipers provided by the SS-OCT software. The Topcon imageNET software was used to automatically calculate the foveal avascular zone (FAZ) area (mm^2^), perimeter (mm), and circularity. The ImageJ software was utilized to measure vessel density on the DCP OCTA macula images. After manually excluding the FAZ area, the OCTA images were binarized using the mean threshold (automatic threshold), and the percentage of white pixels was calculated for the vessel density via the “analyze particle” command^21^. Central and overall nonperfusion areas (NAs) on fluorescein angiography (FA) were measured (Supplementary data 2).

### Statistical analysis

Statistical analysis was performed using Statistical Package for the Social Sciences for Windows (version 24.0; SPSS, Inc., Chicago, IL, USA). One-way analyses of variance (ANOVAs) were used to assess the mean differences between recurrent BRVO, indolent BRVO, and corresponding control quadrants, followed by a post-hoc independent *t*-test for the mean corrected perivascular reflectivity. Bonferroni correction was performed for multiple comparisons. Intergrader reliability was calculated using intraclass correlation coefficient (ICC) analysis. Regression analysis was conducted for several possible factors, including the number of intravitreal anti-VEGF or dexamethasone injections per year, with the corrected mean perivascular reflectivity. To evaluate the effect of disease duration, sub-analysis was performed by dividing the period from the initial diagnosis to the time of OCT and OCTA scan acquisition into two groups (based on 3 years from the initial visit), using the independent *t*-test and regression analysis.

### Ethical approval

Owing to the retrospective nature of image analysis and anonymized data of this study, the need of informed consent procedures was waived by the Institutional Review Board (IRB) of Seoul St. Mary’s Hospital. Waiver of informed consent was granted by the IRB in accordance with the provisions of the IRB. All protocols were also approved by the IRB of Seoul St. Mary’s Hospital, The Catholic University of Korea (KC23RISI0232).

The provisions of IRB: The study corresponds to [waiver of informed consent process] for the following reasons in accordance with relevant domestic and international regulations.

1. Retrospective study of medical record
2. It is practically impossible to obtain consent from the study subject in the course of the study or has a serious impact on the validity of the study
3. There is no reason to estimate the subject's refusal to consent, and even if consent is exempted, the risk to the subject is extremely low.

## Results

**Figure 2:**
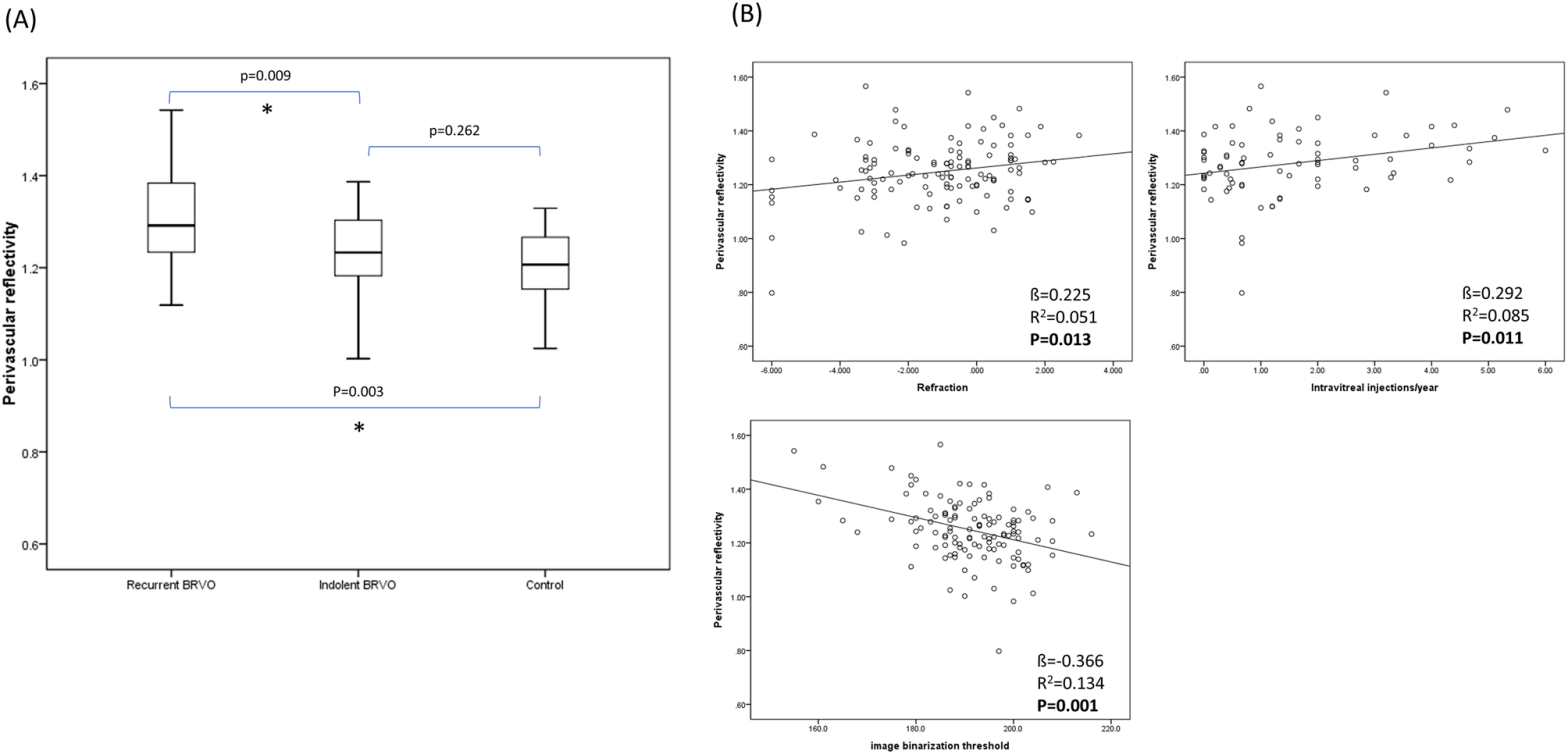
(**a**) The perivascular reflectivity on the en-face optical coherence tomography (OCT) images is presented in the form of box plots. The mean differences between recurrent branched retinal vascular obstruction (BRVO), indolent BRVO, and control quadrants were assessed using the one-way analysis of variance (ANOVA) test, followed by a post-hoc independent *t*-test. The variation of perivascular reflectivity was statistically significant in the one-way ANOVA test (P value < 0.001). The asterisk indicates a statistically significant P-value after Bonferroni correction for multiple comparisons (P < 0.05). (**b**) Factors showing a significant correlation (P < 0.05) with perivascular reflectivity in the multivariate regression analysis are presented as linear regression plots. *β* regression coefficient, *R*^*2*^ coefficient of determination.

Table 1 shows the demographics and characteristics of the study participants, including key measurements in OCT/OCTA for calculating perivascular reflectivity. The ICCs for single measures and average measurements were 0.919 and 0.958, respectively (P < 0.001), indicating excellent agreement between the two graders^22^. In the one-way ANOVA test, the variation in perivascular reflectivity was statistically significant (P < 0.001) in the comparison of recurrent BRVO, indolent BRVO, and corresponding control quadrants. In the post-hoc analysis, the perivascular reflectivity of the recurrent BRVO quadrant was significantly higher than that of indolent BRVO and control quadrants (P = 0.009, 0.003, when Bonferroni correction was applied). There was no significant difference between the indolent quadrants and the control quadrants (Fig. 2a).

**Table 1:** Demographics and characteristics of the study participants.

	Recurrent BRVO (n = 45)	Indolent BRVO (n = 30)	Control (n = 45)	P-value
Age, years	64.60 (± 10.21)	64.30 (± 10.45)	61.97 (± 8.34)	0.386
Sex, male:female	17:28	10:20	15:30	0.888
Disease eye, OD:OS	26:19	21:9	24:21	0.351
Hypertension	40.0 (%)	60.0 (%)	28.8 (%)	**0.026**
Diabetes mellitus	17.8 (%)	20.0 (%)	20.0 (%)	0.957
Intraocular pressure	14.24 (± 3.27)	15.60 (± 3.94)	14.48 (± 2.20)	0.165
Refractive error (spherical equivalent)	− 1.09 (± 1.97)	− 1.75 (± 2.09)	− 0.90 (± 1.94)	0.108
Initial VA (logMAR)	0.336 (± 0.261)	0.251 (± 0.189)	0.048 (± 0.072)	**< 0.001**
Duration to OCT acquisition (months)	34.84 (± 22.67)	28.60 (± 23.12)		0.255
Total number of injections	7.95 (± 7.62)	0.93 (± 0.78)		**< 0.001**
Number of injections per year	2.36 (± 1.47)	0.38 (± 0.36)		**< 0.001**
Initial central macular thickness (CMT)	465.97 (± 155.17)	336.50 (± 158.21)		**0.001**
Sub-foveal choroidal thickness (SFCT)	296.82 (± 81.22)	294.33(± 104.01)		0.912
Deep capillary plexus vessel density in OCTA (%)	45.43 (± 2.56)	46.82(± 2.66)		0.028
Central nonperfusion area (%) on FA	33.11 (± 10.65)	26.01 (± 16.14)		0.158
Central nonperfusion area (D) on FA	4.90 (± 1.70)	3.37 (± 2.32)		**0.045**
Overall nonperfusion area (D) on FA	49.69 (± 41.96)	33.64 (± 33.91)		0.352
FAZ area (mm^2^)	0.37 (± 0.20)	0.38 (± 0.14)		0.803
FAZ perimeter (mm)	2.69 (± 1.10)	3.76 (± 4.20)		0.184
FAZ circularity	0.60 (± 0.10)	0.63 (± 0.12)		0.363
Binarization threshold value	189.93 (± 11.54)	193.30 (± 9.39)	191.26 (± 9.18)	0.377
OCTA HSS area (> 3 consecutive pixels)	1268.28 (± 733.19)	899.63 (± 534.84)	570.60 (± 364.05)	**< 0.001**
OCTA HSS area number	96.04 (± 41.39)	98.03 (± 50.54)	100.55 (± 54.17)	0.908
Mean reflectivity on HSS	139.70 (± 17.56)	141.41 (± 19.75)	148.67 (± 14.50)	**0.037**
Overall en-face OCT mean reflectivity	107.03 (± 17.06)	115.96 (± 15.63)	124.40 (± 8.81)	**< 0.001**
Corrected mean reflectivity on HSS	1.31 (± 0.09)	1.22 (± 0.13)	1.19 (± 0.07)	**< 0.001**

*VA* visual acuity, *HSS* high-signal strength, *FA* fluorescence angiography, *D* disc diameter, *FAZ* foveal avascular zone.

Corrected mean reflectivity was calculated as the mean reflectivity on the HSS area/overall en-face OCT mean reflectivity.

Data are expressed as mean (± SD) or frequency, as appropriate.

Two-sided *P*-values < 0.05 were considered to indicate statistical significance.

Statistically significant P-values are highlighted in bold.

**Figure 3:**
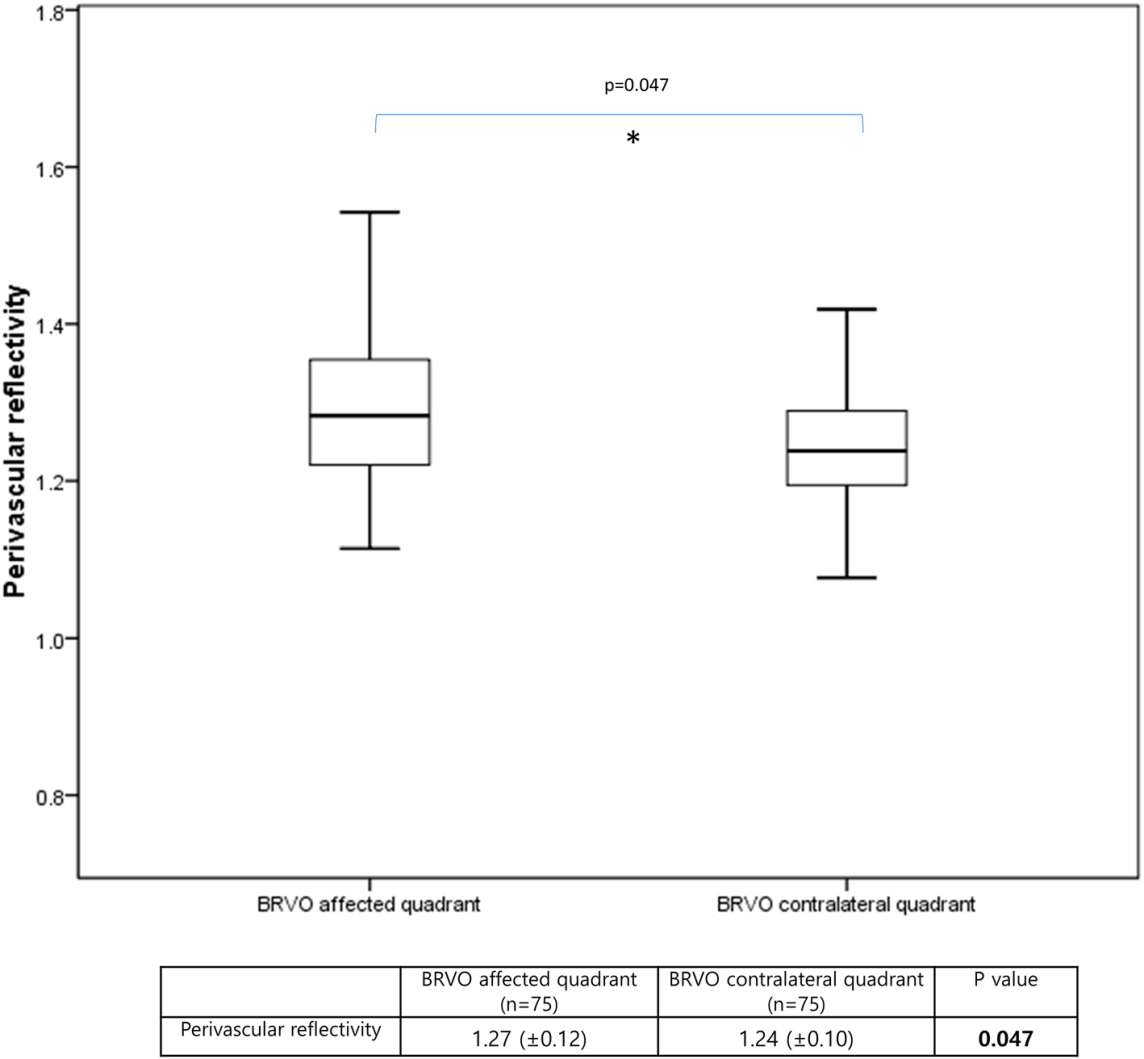
The mean difference in perivascular reflectivity between BRVO obstructive and contralateral quadrants in the overall BRVO group (n = 75). Using paired *t*-test, the reflectivity was measured significantly higher in obstructive quadrants than in contralateral quadrants (P = 0.047).

The results of the linear regression analysis of several potential factors influencing perivascular reflectivity are presented in Table 2. Age, intraocular pressure, refractive errors, the image binarization threshold value, and the average number of intravitreal injections per year demonstrated significant correlations with perivascular reflectivity in the univariate analysis (P = 0.008, 0.015, 0.013, < 0.001 and 0.011, respectively; Fig. 2b). In the multivariate regression analysis, refractive errors, the image binarization threshold value and the average number of intravitreal injections per year demonstrated significant P-values (P = 0.041. 0.005 and 0.007, respectively). The mean difference in perivascular reflectivity between BRVO obstructive and contralateral quadrants in the overall group (n = 75) was analyzed with paired *t*-test (Fig. 3). The reflectivity was significantly higher in the affected quadrants than in the contralateral quadrants (P = 0.047). The sub-analysis, based on disease duration, revealed no significant differences in perivascular reflectivity and the average number of injections per year between the two groups. However, the linear regression analysis revealed that the < 3 years group exhibited a significant correlation between reflectivity and the average number of injections (P = 0.021; Table 3).

**Table 2:** Linear regression analysis of factors associated with perivascular reflectivity on en-face OCT in BRVO.

	Perivascular reflectivity			
	Univariate		Multivariate	
	Standardize β	P-value	Standardize β	P-value
Sex (M/F)	− 0.030	0.748		
Age	0.239	**0.008**	0.026	0.830
OD/OS	0.094	0.305		
Hypertension	0.012	0.893		
Diabetes mellitus	− 0.023	0.804		
IOP (mmHg)	− 0.221	**0.015**	− 0.059	0.595
Refractive errors (SE)	0.225	**0.013**	0.256	**0.041**
BCVA, logMAR	0.141	0.123	− 0.192	0.092
Image binarization threshold	− 0.366	**< 0.001**	− 0.412	**0.005**
Average number of intravitreal injections per year	0.292	**0.011**	0.304	**0.007**
Duration between the initial visit and OCT acquisition (months)	0.002	0.983		
SFCT (μm)	0.019	0.869		
CMT (μm)	0.025	0.832		
Vessel density (%)	− 0.171	0.143	0.194	0.172
Central nonperfusion area (%) on FA	0.005	0.980		
Central nonperfusion area (D) on FA	− 0.123	0.509		
Overall nonperfusion area (D) on FA	− 0.146	0.433		
FAZ area (mm^2^)	− 0.004	0.975		
FAZ perimeter (mm)	− 0.077	0.513		
FAZ circularity	0.044	0.705		

*β* regression coefficient, *BCVA* best-corrected visual acuity, *logMAR* logarithm of the minimum angle of resolution, *IOP* intraocular pressure, *CMT* central macular thickness, *FA* fluorescence angiography, *D* disc diameter, *FAZ* foveal avascular zone, *SFCT* subfoveal choroidal thickness, *SE* spherical equivalent.

Two-sided *P*-values < 0.05 were considered to indicate statistical significance. Statistically significant P-values are highlighted in bold.

Factors with P values < 0.2 in the univariate analysis were included in the multivariate analysis.

**Table 3:** Sub-analysis of differences in perivascular reflectivity and number of injections according to disease duration.

	Duration < 3 years n = 45	Duration > 3 years n = 30	P-value
	Mean (± SD) Standardize β/R^2^/P-value	Mea n(± SD) Standardize β/R^2^/P-value	
Duration from the initial visit to OCT acquisition (months)	17.64 ± 8.16	56.43 ± 20.60	**P < 0.001**
Overall number of intravitreal injections	2.84 ± 2.57	8.60 + 9.41	**P = 0.003**
Mean difference in reflectivity	1.27 ± 0.14	1.28 ± 0.08	P = 0.813
Mean difference in intraocular injections/year	1.64 ± 1.52	1.47 ± 1.53	P = 0.654
Regression between reflectivity and injections/year	β = 0.342/R^2^ = 0.117/**P = 0.021**	β = 0.203/R^2^ = 0.041/P = 0.283	

The difference in reflectivity and the number of injections between the two groups divided by the period from the first visit to obtaining OCT.

The correlation between reflectivity and the number of injections was analyzed in each group.

Statistically significant P-values are highlighted in bold.

*β* regression coefficient, *R*^*2*^ coefficient of determination.

Two-sided P-values < 0.05 were considered to indicate statistical significance.

Data are expressed as mean (± SD).

## Discussion

The mean perivascular reflectivity of the quadrant in which vascular occlusions occurred in the BRVO group was significantly higher than that in the indolent and control groups. This can be explained by four hypotheses. First, the degree of reflection may increase owing to the disorganization of the perivascular tissue due to vascular occlusion. Previous reports of correlations between increased reflectivity of the inner retina in retinal vascular obstruction and visual outcomes have suggested a similar pathophysiology^11,12,23^. Second, reflectivity may increase owing to an upsurge in local inflammatory cells triggered by ischemia. Studies have suggested that perivascular macrophages and microglia are retinal hyper-reflective foci, and we speculate that if a large number of these inflammatory cells are scattered around blood vessels, the reflectivity increases progressively^24,25^. Third, glial cell proliferation may occur during the remodeling process following the infiltration of perivascular inflammatory cells, and the surrounding tissue may undergo mesenchymal transition into fibrotic tissue after a certain period. This process may be similar to that of hyper-reflectivity observed in epiretinal membranes^26,27^. Lastly, perivascular reflectivity may increase owing to the higher density of enlarged collateral vessels per unit area, and congested large vessels are likely to be widely distributed in the obstructive region. Studies on the reflectivity of vessels on OCT and OCTA indicate that the higher the vessel size, the more direct the increase in reflectivity^28,29^. In the regression analysis conducted in the present study, the correlation between vessel density and perivascular reflectivity was not statistically significant. However, wide OCTA HSS area and low overall en-face OCT mean reflectivity in the recurrent BRVO group demonstrated that congested vessels with dark surroundings, which may arise from edematous stroma or the ischemic area, would increase the corrected mean perivascular reflectivity calculated in the present study. The significant association between the image binarization threshold and perivascular reflectivity is consistent with the congestion vessel-related process rather than just the ischemic process. As the image binarization threshold was set low, a larger OCTA HSS area would be selected, finally elevating perivascular reflectivity.

The significant difference in perivascular reflectivity between the BRVO obstructive and contralateral quadrants may indicate that changes in the perivascular tissue occur mainly at the obstruction site. The degree of reflectivity changes becomes insignificant in regions farther away from the obstructive area.

Both the univariate and multivariate regression analyses showed that the average number of intraocular injections significantly affected perivascular reflectivity. Considering this, we suggest that perivascular change, which may come from perivascular tissue disorganization or congested vessel with surrounding edematous stroma we hypothesized above, has an important effect on the recurrence of clinical BRVO ME and can be used as an indicator to predict recurrence, despite the timing of OCT scan acquisition varying among patients. Moreover, we believe that these results could serve as a foundation for developing appropriate treatments to prevent the recurrence of BRVO ME, provided that large-scale well-designed studies and animal studies that present a clear pathophysiology are carried out. Regarding refraction, the alteration of reflectivity measurement may be attributed to the axial length, and despite correcting the mean reflectivity on HSS by dividing it by the total en-face OCT mean reflectivity, refractive error still had a significant impact on the reflectivity measurement.

Macular vessel density and diameter on OCTA are related to BRVO ME recurrence^6,30,31^. A possibility cannot be ruled out that the reduction rate in the macular vessel density and degree of macular ischemia altered perivascular tissue composition, increasing the reflectivity. However, considering that the reflectivity measurement was not correlated with the vessel density, FAZ parameters, or nonperfusion area on FA, another pathophysiology, regardless of macular ischemia, might be involved. The association of the central nonperfusion area on FA not with perivascular reflectivity but with the number of intravitreal injections demonstrated that ischemia and increased reflectivity from another pathophysiology would be independent risk factors for BRVO ME recurrence. Perivascular reflectivity could be a good biomarker for vessel congestion and perivascular tissue disorganization. An in-depth study on the relationship between macular microvasculature and perivascular reflectivity would elucidate the pathophysiology of clear BRVO ME recurrence.

After conducting a sub-analysis, wherein the period from the initial examination to the time of OCT/OCTA acquisition was divided into two groups (based on 3 years from the initial visit), the difference between the two groups was not statistically significant. Therefore, disease duration and the total number of injections did not have a significant impact on reflectivity measurements. However, perivascular reflectivity was significantly correlated with the average number of injections per year only in the < 3 years (from the initial visit) group. Therefore, if the OCT/OCTA images are used to determine the possibility of recurrences, they must be analyzed as soon as the ME is resolved. In a multicenter study by Costa et al., half of the patients treated with anti-VEGF or corticosteroid intravitreal injections for RVO showed complete resolution of macular edema 3 years after the diagnosis^32^. In several studies, intensified injection therapy was administered during the first year of treatment, and remission was partially achieved while gradually reducing the interval^5^. Therefore, the authors judged that setting the standard at 3 years would be more suitable for evaluating the effect of disease duration on reflectivity rather than frequent injections on reflectivity.

A limitation of this study is the absence of an academic background required to fully comprehend the precise mechanism that increases perivascular reflectivity. To verify the hypotheses presented above, additional animal or experimental studies should be conducted. Although there was no statistically significant change in reflectivity according to the disease duration, prospective studies controlling such factors are desirable because the total observation period when calculating the average number of injections and the timing of OCT/OCTA analysis vary. The length of the prevalence period affects the extent to which the exudates deposited in the macula impact the reflectivity measurement. Another limitation was its suitability as a threshold of mean + 2SD in the OCTA image binarization. The HSS setting of an area with a pixel value of mean + 2SD or higher in this study made analyzing the vessel areas with fewer signals impossible in OCTA scans. A method for comparing RPE signal strengths as a threshold was introduced in studies on hyperreflective foci and could be applied alternatively^33,34^. In addition, the accuracy of the analysis may be reduced by the variability of the image quality or the extent to which the fovea is located centrally.

In conclusion, despite the aforementioned limitations, we believe that this study is of considerable significance as a pioneering investigation, presenting perivascular reflectivity on en-face OCT as a prognostic marker for the recurrence of BRVO ME. These novel findings may eventually be applied to assist clinicians and researchers with understanding the pathophysiology of ME recurrence.

### Supplementary Information

Supplementary Information 1.

Supplementary Information 2.

## Data Availability

All data generated or analyzed during this study are included in this published article (and its Supplementary Information files).
