# Supplementary figures and images for: Alteration of perivascular reflectivity on optical coherence tomography of branched retinal vein obstruction
Source: Sci Rep. 2023 Sep 22;13:15847. doi: 10.1038/s41598-023-41691-4 (PMC10517127; doi:10.1038/s41598-023-41691-4)

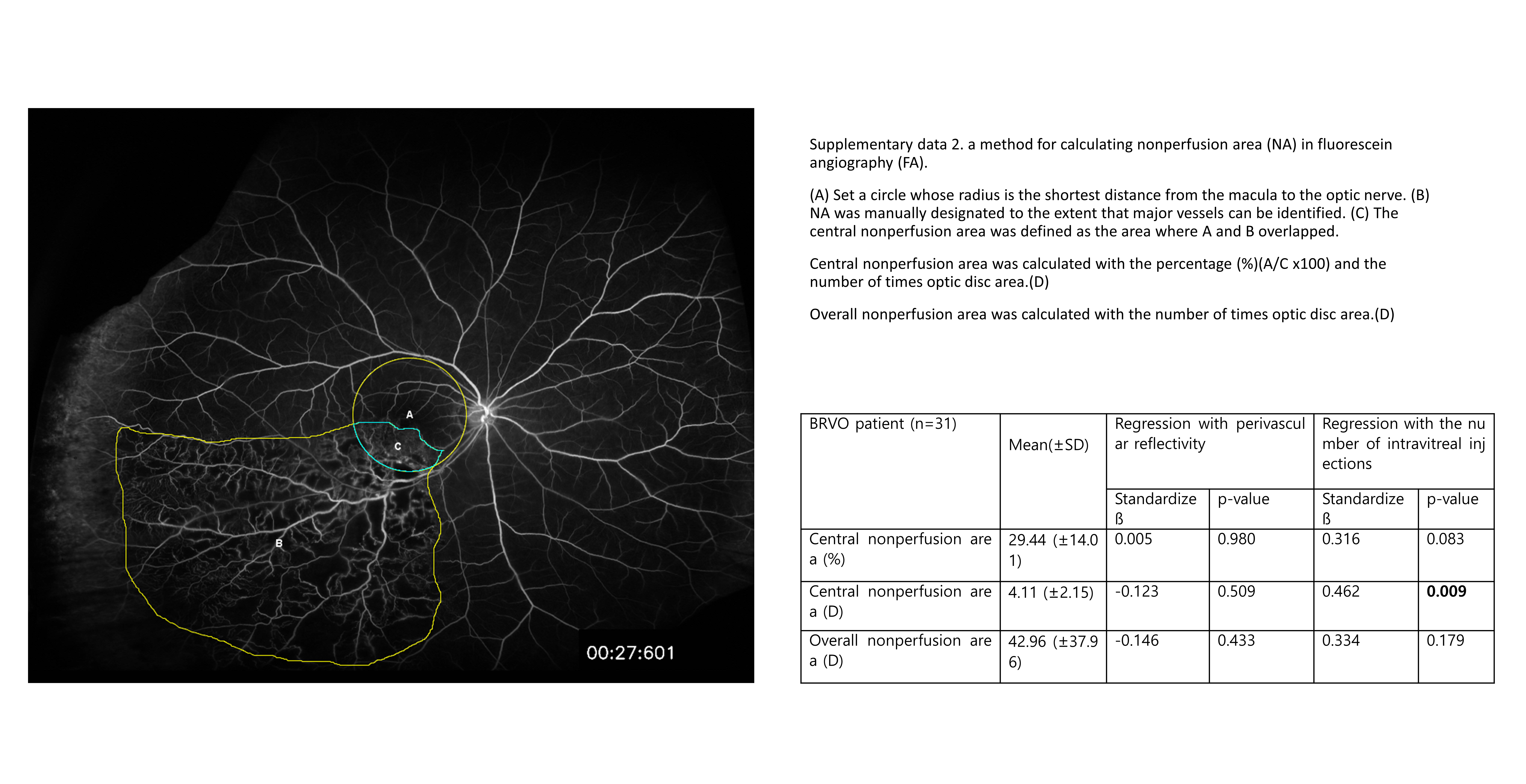

Supplement: Supplementary file 2 — Supplementary Information 2. [file 41598_2023_41691_MOESM2_ESM.tif]
